# Implementation of the meaningful activity for managing behavioral symptoms of distress (MAC‐4‐BSD) intervention in assisted living

**DOI:** 10.1002/alz.086604

**Published:** 2025-01-09

**Authors:** Susan Scherr

**Affiliations:** ^1^ Stevenson University, Owings Mills, MD USA

## Abstract

**Background:**

Most assisted living (AL) settings organize and provide opportunities for residents to participate in activities (e.g., exercise, music, arts and craft, cognitive activities, religious services, community outings). Despite the known benefits of activity participation, it is widely reported that there is a lack of activities that are considered meaningful for residents and thus many residents spend much of their time during the day disengaged. Further, there are numerous challenges to engaging assisted living (AL) residents with dementia in meaningful activity at the resident and community level. Resident challenges include acute medical events, advanced age, comorbidities, physical and cognitive abilities, lack of motivation, cultural expectations, pain, and behavioral symptoms of distress (BSD). Community level challenges include environments and policies that limit opportunities for meaningful activities and limited resources.

**Method:**

To overcome these challenges and optimize engagement in meaningful activity for residents with dementia, the Meaningful Activity for Managing Behavioral Symptoms of Distress (MAC‐4‐BSD) intervention was developed. MAC‐4‐BSD is theoretically based using the Social Ecological Model and Social Cognitive Theory and includes four components: 1. Assessment of the environment and policies; 2. Education of staff; 3. Assessment of resident preferences and goals for meaningful activity; 4. Mentoring and motivating staff and residents. The overall aim of MAC‐4‐BSD is to reduce BSD, increase engagement in meaningful activity, and improve quality of life for residents by modifying the environment and empowering staff to facilitate and engage residents with dementia in AL settings. The MAC‐4‐BSD intervention is implemented by a Research Facilitator working with a facility champion and monthly meetings take place with a facility stakeholder team to provide updates on successful strategies and brainstorm challenges encountered.

**Results:**

To date, a total of 5 AL settings and 71 residents with dementia have participated in the study. Based on content analysis of field notes we will describe stakeholder goals established, barriers to implementation, and facilitators to meet goals in the participating settings.

**Conclusions:**

Key findings include the influence of administrative or leadership support, level of engagement among direct care staff, interdisciplinary collaboration, and access to resources and supplies in the environment to facilitate meaningful activity for residents with dementia.

